# Cemiplimab for advanced cutaneous squamous cell carcinoma in kidney transplant recipients

**DOI:** 10.3389/fneph.2022.1041819

**Published:** 2022-10-31

**Authors:** T. Van Meerhaeghe, J.F. Baurain, O. Bechter, C. Orte Cano, V. Del Marmol, A. Devresse, P. Doubel, M. Hanssens, R. Hellemans, D. Lienard, A. Rutten, B. Sprangers, A. Le Moine, S. Aspeslagh

**Affiliations:** ^1^ Department of Nephrology, Hôpital Erasme – Université Libre de Bruxelles (ULB), Brussels, Belgium; ^2^ Department of Oncology, Clinique Universitaire Saint-Luc – Université Catholique de Louvain (UCLouvain), Brussels, Belgium; ^3^ Department of Oncology, Universitair Ziekenhuis (UZ) Leuven – Katholieke Universiteit Leuven (KUL), Leuven, Belgium; ^4^ Department of Dermatology, Hôpital Erasme – Université Libre de Bruxelles (ULB), Brussels, Belgium; ^5^ Department of Nephrology, Clinique Universitaire Saint-Luc – Université Catholique de Louvain (UCLouvain), Brussels, Belgium; ^6^ Department of Nephrology, Academisch Ziekenhuis (AZ) Groeninge, Kortrijk, Belgium; ^7^ Department of Oncology, Academisch Ziekenhuis (AZ) Groeninge, Kortrijk, Belgium; ^8^ Departement of Nephrology, Universitair Ziekenhuis (UZ) Antwerpen, Antwerpen, Belgium; ^9^ Department of Oncology, GasthuisZuster, Antwerpen, Belgium; ^10^ Department of Nephrology, Universitair Ziekenhuis (UZ) Leuven – Katholieke Universiteit Leuven (KUL), Leuven, Belgium; ^11^ Department of Oncology, Universitair Ziekenhuis (UZ) Brussel – Vrije Universiteit Brussel (VUB), Brussels, Belgium

**Keywords:** cemiplimab, kidney transplant recipients, cutaneous squamous cell carcinoma, immune checkpoint inhibitors, rejection

## Abstract

**Background:**

Kidney transplant recipients (KTR) are at increased risk of cancer due to chronic immunosuppression. Non-melanoma skin cancer has an excess risk of approximately 250 times higher than the general population. Moreover, in solid organ transplant recipients (SOTR) these cancers have a more aggressive behavior, with an increased risk of metastasis and death. Cemiplimab, a human monoclonal IgG4 antibody against programmed cell death (PD-1) has shown considerable clinical activity in metastatic and locally advanced cutaneous squamous cell carcinoma (cSCC) in patients for whom no widely accepted standard of care exists. Cemiplimab has therefore been approved since 2018 for the treatment of advanced cSCC. However, data regarding the use of cemiplimab in SOTR and particularly in KTR are scarce and based on published case reports and small case series. In this study, we report on the real-life outcome of cemiplimab use in a Belgian cohort of seven KTR suffering from advanced cSCC.

**Objective:**

To report on the overall response rate (ORR) and safety of cemiplimab in KTR in Belgium.

**Results:**

Seven patients suffering from advanced cSCC, treated with cemiplimab, between 2018 and 2022, in Belgium were identified. Three patients were on corticosteroid monotherapy, one patient on tacrolimus monotherapy and three patients were on at least 2 immunosuppressants at start of cemiplimab. The ORR was 42.8%, stable disease was seen in 14.3% and progressive disease was found in 42.8% of the patients, respectively. The median administered number of cycles was 12, interquartile range (IQR) 25-75 [3.5 – 13.5]. All patients were treated with surgery before administration of cemiplimab, 71.4% received additional radiotherapy and only 1 patient was treated with chemotherapy prior to receiving cemiplimab. Biopsy-proven acute renal allograft rejection was observed in one patient, who eventually lost his graft function but showed a complete tumor response to treatment. Low grade skin toxicity was seen in one patient of the cohort.

**Conclusion:**

The present case series shows that the use of cemiplimab in KTR with advanced cSCC who failed to respond to previous surgery, chemo – and/or radiotherapy treatment is associated with an ORR of 42.8% with minimal risk of graft rejection (14.3%) and good tolerance.

## Introduction

Malignancy is a significant adverse event in kidney transplant recipients (KTR). The overall risk of developing cancer has been reported to be 2 - and 4 – fold higher compared to the general population (1, 2). Some cancer types are overrepresented in KTR, especially non-melanoma skin cancer (NMSC) with an excess risk of approximately 250 times higher than the general population (3). The most frequent NMSC encountered in KTR is cutaneous squamous cell carcinoma (cSCC). Moreover, KTR patients tend to have a more aggressive behavior of the disease and an increased risk of metastasis and cancer related death (4, 5). cSCC tumors harbor a high mutation burden, which has been associated with good response to immune checkpoint inhibitors (6). Cemiplimab, a human monoclonal IgG4 antibody against anti-PD-1 has shown favorable overall survival and progression free survival in immunocompetent patients suffering from advanced cSCC (7–9). However, these trials excluded patients with a history of solid organ transplantation owing to concerns about alloimmunity, organ rejection, and the use of concomitant immunosuppressive therapy possibly abolishing the efficacy of immunotherapy. Evidence on efficacy of immune checkpoint inhibitors (ICI) in KTR is mainly based on case reports, case series and a few systematic reviews reporting on its postmarketing use (10–17).

Herein, we review the real-world experience with cemiplimab in KTR for advanced cSCC in Belgium.

## Patients and methods

Kidney transplant recipients, who presented an advanced cSCC for which they were treated with cemiplimab between 2018 and first of January of 2022 in Belgium were included in the present study. Advanced cSCC was defined as either locally advanced disease not amenable to curative surgery or radiotherapy (RT) or metastatic disease. cSCC stage was defined according to the American Joint Committee on Cancer (AJCC) TNM Staging system for cSCC of Head and Neck (18). Immune response was evaluated according to the iRECIST criteria for solid tumors (19). Objective response rate (ORR) was defined as complete response (CR) or partial response (PR). Seven patients were identified across 5 different centers. Clinical and demographic features, immunosuppression, allograft function, efficacy and outcomes were reviewed. Central and Local ethics’ committees of the different centers involved, approved the study.

## Statistics

Data are described using mean ± standard deviation (SD) or medians and interquartile ranges (IQR) 25-75 depending on the distribution of the data. Estimates of overall survival (OS, the time from introduction of cemiplimab to death due to any cause) was assessed by the Kaplan-Meier method for the 7 patients. Statistical analysis was done by using MedCalc Software Ltd.

## Results

### Baseline characteristics

A total of 7 KTR treated with cemiplimab from 5 different centers in Belgium were identified between 2018-2022 in Belgium. **Table 1** illustrates baseline characteristics of included patients. All patients but one patient suffered from stage IV cSCC according to the AJCC and one patient had a locally advanced cSCC with eye localization, which was not treatable neither with surgery nor with radiotherapy. The mean age of the patients was 68.1 ± 10.5 years and most patients were male (71.4%). The mean body mass index (BMI) of the patients was 23.8 ± 3.9 kg/m^2^ and the median Karnofsky score of the patients was 80 (IQR 25-75 [65-90]). All patients were dialyzed prior to renal transplantation. The etiology of the end-stage kidney disease was diverse: genetic in 3, vascular in 1, tubulo-interstitial disease in 1, chronic pyelonephritis in 1 and glomerulonephritis in 1. No patient was suffering from an underlying auto-immune disorder (see **Table 1**).

**Table 1:** Patients’ characteristics and main outcomes.

	Kidney transplant recipients n = 7, *n* (%)
Age (mean ± *SD*) (in years)	68.1 ± 10.5
Male sex	5 (71.4)
BMI (mean ± *SD*) (in kg/m^2^)	23.8 ± 3.9
Karnofsky status (median IQR 25-75)	80 (65-90)
Causes of ESRD	
Genetic	3 (42.8)
Vascular	1 (14.3)
Tubulo-interstitial	1 (14.3)
Chronic pyelonephritis Glomerulonephritis	1 (14.3) 1 (14.3)
Presence of diabetes	2 (28.6)
Presence of hypertension	5 (71.4)
Active smoking	1 (14.3)
Dyslipidemia	5 (71.4)
Concomitant medication use:	
NSAID	1 (14.3)
PPI	4 (57.1)
Antibiotic use	1 (14.3)
Baseline eGFR CKD-EPi (mean ± *SD*)	60.4 ± 24.5 ml/min
Number of HLA mismatches (median IQR 25-75) *	3 (1-3)
Type of donor	
Deceased kidney donor	7 (100)
Living kidney donor	0
History of acute rejection	None
Presence of DSA	None
Immunosuppression	
CTC alone	3 (42.8)
TAC ALONE	1 (14.3)
CTC + MMF + mTORi	1 (14.3)
CTC + Cyclosporine	1 (14.3)
CTC + TAC + mTORi	1 (14.3)
Prior treatments	
Surgery	7 (100)
Systemic therapy	1 (14.3)
Radiotherapy	5 (71.4)
Time from transplant to ICI initiation (median IQR 25-75) in years	10.6 (6.1 – 19.9)
Cycles of CEMIPLIMAB (median IQR 25-75)	12 (3.5-13.5)
Tumor response CR	2 (28.6)
PR	1 (14.3)
SD	1 (14.3)
PD	3 (42.8)
Immune related adverse events	1 (14.3)
Deceased	4 (57.1)
related to tumor progression	3 (42.8)
related to rejection	0
other	1 (14.3)

*Information missing for 2 patients.

SD, standard deviation; BMI, body mass index; NSAID, non-steroidal anti-inflammatory drugs; PPI, proton pump inhibitors; eGFR, estimated glomerular filtration rate; DSA, donor specific antibodies; HLA, human leukocyte antigen; CTC, corticosteroids; TAC, tacrolimus; MMF, mycophenolate mofetil, mTORi mammalian target of rapamycin inhibitors; CR, complete response; PR, partial response; SD, stable disease; PD, progressive disease.

Diabetes was seen in 28.6%, arterial hypertension in 71.4%, active smoking in 14.3% and dyslipidemia in 71.4% of the patient population.

Mean baseline estimated Glomerular Filtration Rate (eGFR) according to Chronic Kidney Disease Epidemiology Collaboration (CKD-EPI) was 60.4 ± 24.5 ml/min. No patient in the study had donor specific antibodies and none had a history of acute rejection. Median HLA mismatches were 3 (IQR 25-75 [1-3], but information was lacking for 2 patients in the study. All patients received a deceased donor kidney. Immunosuppression was lowered prior to introduction of cemiplimab in 6 out of 7 patients. Three patients (42.8%) were treated with low dose methylprednisolone, 1 patient was on tacrolimus monotherapy (14.3%), and 3 patients (42.8%) received 2 or more than 2 immunosuppressive drugs. Prior to introduction of cemiplimab, all patients were treated with surgery, 1 patient had chemotherapy (14.3%) and 5 patients (71.4%) received radiotherapy. The median time from transplantation to cemiplimab initiation was 10.6 years (IQR 25-75 [6.1-19.9]). The median cycles administered of cemiplimab were 12 (IQR 25-75 [3.5-13.5]).

### Tumor response rate

**Figure 1:**
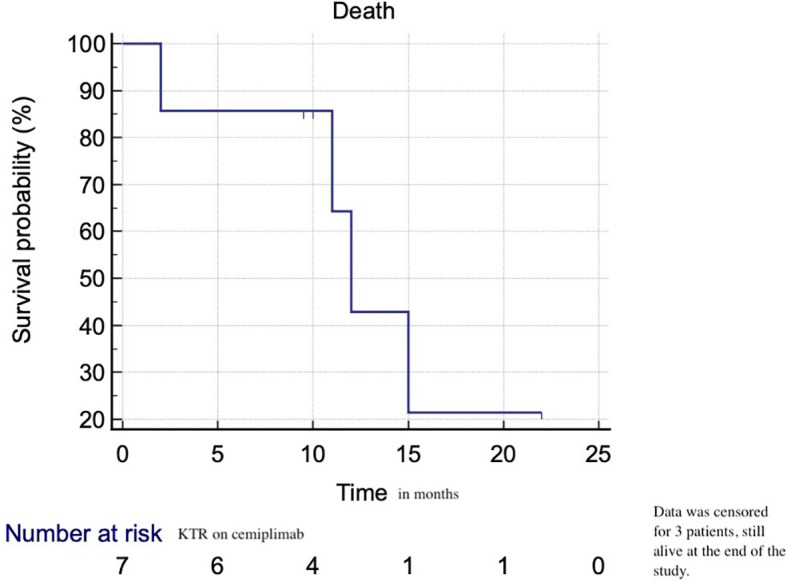
Overall survival of KTR on cemiplimab for advanced cSSC.

ORR was seen in 3 patients (42.8%), stable disease (SD) in 1 (14.3%) and progressive disease in 3 (42.8%). Death due to tumoral progression was seen in 3 patients (42.8%) (**Table 2**). The median overall survival (OS) estimated by the Kaplan Meier was 12 months (95% CI of the median 2 – 15 months) (**Figure 1**).

**Table 2A:** Details of the characteristics and outcomes of the patients included in the study.

Patient	Sex/age (years)	Number of kidney transplantations	Karnofsky status	Cause of ESKD	Baseline eGFR according to CKD-Epi in mL/min/1.73m^2^	Number of HLA mismatches	cSCC stage – site of metastasis
1	M/75	1	80	vascular	71	2	T2NxM1 – skin and lymph nodes
2	F/64	2	90	genetic	61	3	TxNxM1 – lung and lymph nodes
3	F/72	1	70	genetic	42	NA	T3NxM1 – lymph nodes
4	M/80	1	60	tubulo-interstital	86	5	T3NxM1 – lung, bone and lymph nodes
5	M/59	1	60	chronic pyelonephritis	31	NA	TxNxM1 – lymph node and skin
6	M/51	3	90	genetic	94	1	T3N0M1 – bone and parotid
7	M/76	1	100	glomerulonephritis	38	3	T1NxM0 – eye localisation

M, male; F, female, ESKD: end-stage kidney disease; eGFR, estimated glomerular filtration rate; CDK-Epi, HLA, human leucocyte antigen; cSCC, cutaneous squamous cell carcinoma; NA, not applicable.

**Table 2B:** Details of the characteristics and outcomes of the patients included in the study.

Previous chemotherapy	Previous Radiotherapy	Time since Tx and start of cemiplimab in years	Immunosuppression at start of cemiplimab	Number of cemiplimab infusions	Allograft rejection	RECIST criteria	rAE	Outcome – cause of death
No	Yes	6.4	CTC 8 mg	3	Yes – acute TCMR Banff grade IIA	No	T2NxM1 – skin and lymph nodes	Death – COVID-19 infection
No	Yes	3.6	CTC 5 mg, trough TAC level 2.9 ng/ml, trough mTORi level 5 ng/ml	13*	No	No	TxNxM1 – lung and lymph nodes	Alive
No	No	19.4	CTC 5 mg,trough cyclosporine level 96 ng/ml	4	No	No	T3NxM1 – lymph nodes	Death – tumoral progression
No	Yes	10.6	CTC 4 mg,MMF 500 mg,mTORi trough level 3 ng/ml	17	No	No	T3NxM1 – lung, bone and lymph nodes	Death – tumoral progression
Yes	Yes	30.4	Trough level TAC 5 ng/ml	14*	No	Grade I-II skin toxicity	TxNxM1 – lymph node and skin	Alive
No	No	5.8	CTC 8 mg	12	No	No	T3N0M1 – bone and parotid	Death – tumoral progression
No	No	20.4	CTC 8 mg	2	No	No	T1NxM0 – eye localisation	Alive

Tx, transplantation; iRECIST, immune response evaluation criteria in solid tumors; CR, complete response; PR, partial response; SD, stable disease; PD, progressive disease; TCMR, T-cell mediated rejection; CTC, corticosteroids; TAC, tacrolimus; mTORi, mammalian target of rapamycine inhibitor. *ongoing treatment.

In patients with a CR or PR response to cemiplimab, 1 patient suffered from a difficult to treat eye localization, and 2 patients had lymph node and skin metastasis. There was no involvement of other organs or bone lesions. On the contrary 2 of the 3 patients with progressive disease (PD) had lung and/or bone lesions. Patients on monotherapy (CTC or TAC alone) tended to have a better tumoral response compared to patients with at least two immunosuppressive treatments.

### Safety of cemiplimab

Only one patient (14.3%) developed biopsy-proven acute graft rejection during his treatment with cemiplimab within 2 weeks after administration of the first dose. He was on corticosteroid monotherapy at the time of rejection. Histopathological analysis showed acute T-cell mediated rejection Banff grade IIA. Treatment consisted of high dose glucocorticosteroids and subsequent transplantectomy for life-threatening rupture of his graft. He showed however a complete tumor response (**Table 2**).

IrAEs other than graft rejection occurred only in one patient of the entire cohort and consisted of a grade I-II skin toxicity, without need to withhold immunotherapy.

## Discussion

The present case series shows that in KTR with advanced cSCC, who failed to respond to surgery, chemo- and/or radiotherapy, cemiplimab is associated with a good ORR of 42.8% and a low risk of graft rejection (14.3%). Indeed, the two major concerns regarding the use of immune checkpoint inhibitors in solid organ transplant recipients (SOTR) are the risk of allograft rejection and the potential reduction in the anti-cancer response related to the concomitant immunosuppression. Anti-PD-1, anti-PD-L1 and anti-CTLA-4 based therapies activate the immune system, causing the expansion and activation of various immune cell subsets. These changes in immunity can enhance anti-tumor response but also autoimmunity and alloimmunity, therefore increasing the risk of allograft rejection. Due to the fact that patients with a history of organ transplant and with concomitant immunosuppressive therapy are almost always excluded from immunotherapy trials, data on the efficacy of ICI in SOTR are scarce.

One case series of 7 patients, including kidney, liver and lung transplant recipients suffering from advanced cSCC treated with anti-PD-1, showed an ORR of 57.1% (14). Another multicenter study including 69 KTR reported an ORR of 36% in the subgroup of patients suffering from cSCC, with a significantly prolonged survival with the use of ICI (median overall survival 19.8 months vs. 10.6 months) (13). The efficacy of ICI in cSCC was further confirmed in a larger systematic review, showing that SOTR suffering from cSCC derived the most clinical benefit from treatment with ICI compared to other cancer types with an ORR of 68.2%. Our data are in line with these findings (17).

The Kaplan-Meier estimate of the median survival was 12 months in our study and data were censored for 3 patients (still alive at the end of the study). We do not have a control KTR population treated with standard therapy to compare our results. An integrated analysis of a phase 2 study of cemiplimab in advanced cSCC in patients without SOTR, the Kaplan-Meier estimated probability of OS was 73.3% (95% CI: 66.1% to 79.2%) at 24 months, with median OS not reached (20). Whether OS on cemiplimab is prolonged in SOTR suffering from advanced cSCC needs to be addressed in further prospective trials.

The main concern of the usage of ICI in KTR is graft rejection. Cancer after transplantation is mostly managed by a reduction in immunosuppression and with the addition of ICI this might trigger allograft rejection (1). In most case reports and case series published so far, the highest rejection rates were identified in KTR (around 42-44%) compared to other SOTR (10–17). Factors associated with graft rejection were a history of acute rejection, anti-PD-1 usage and single agent immunosuppressive treatment. However, treatment with at least one other IS than corticosteroids, usage of mechanistic target of rapamycine inhibitors (mTORi) and longer time after transplantation (> 8 years) was associated with lower risk of rejection (10–17). Graft rejection in the study of Murakami et al. was associated with high comorbidity as 65.5% of the patients lost their graft function after rejection (13). The patient who rejected his graft in our case series was on low dose methylprednisolone and developed rejection within 2 weeks after his first infusion of cemiplimab, in line with the timing seen in other published reports, where the median time to rejection was 21 days. Despite rescue treatment with high dose glucocorticosteroids patient lost his graft function, but achieved a complete tumor response. The rejection rate in our study is particularly low, compared to published reports. Factors associated with this low rejection rate could not be identified considering the small number of patients included in the study. Nonetheless, the use of cemiplimab in this patient population to control tumor burden is encouraging. Indeed, cemiplimab seems to be associated with lower rates of graft rejection compared to pembrolizumab and nivolumab (13, 16). This observation could be due to different cancer types treated with pembrolizumab and nivolumab in the different case series.

It is well known that mTORi have anti-proliferative effects and thus may potentially prevent allograft rejection (21). Data reported by Murakami et al. and Portuguese et al., show that KTR on mTORi tend to reject less compared to patients under other immunosuppressive regimens. Two patients in our study on mTORi did not present with allograft rejection. A recent multicenter, single arm, phase I study in KTR with cancer showed that addition of nivolumab without preventive reduction in immunosuppression did not impede on tumor response and reduced rejection rates to 12% (22). The patients enrolled in the study were at low immunological risk with stable graft function, had baseline low immunosuppression and suffered from a variety of cancers. These factors might have biased the results, but this is the first prospective study in KTR and is important to consider when confronted with the decision to lower immunosuppression before starting immunotherapy. Future studies need to address which immunosuppressive regimen is the most feasible to combine with ICI therapy. Two prospective trials are underway to specifically explore the efficacy and safety of ICI in KTR, including the “Tacrolimus, Nivolumab, and Ipilimumab in Treating Kidney Transplant Recipients with Selected Unresectable or Metastatic Cancers” (NCT03816332) and the “Cemiplimab in AlloSCT/SOT Recipients with CSS (CONTRAC)” (NCT04339062) (23, 24). One could indeed speculate that there is a therapeutic window for these patients where immunosuppressive therapy is least interfering with the therapeutic benefit of anti-PD-1 based therapy but is also effectively preventing graft rejection.

Well known side effects of immune checkpoint inhibitors are the development of immune related adverse events. In the case series of Tsung et al., 2 of the 7 SOTR developed irAE and one case study showed severe life-threating pneumonitis after cemiplimab treatment for advanced cSCC (14, 25). In our patient cohort only one patient developed low-grade skin toxicity without the need to withhold cemiplimab, with an ongoing tumor response to cemiplimab. Ideally, predictive biomarkers of graft rejection and tumoral response in KTR need to be identified, to better select those patients that may benefit from ICI without compromising their graft during the administration of ICI. One study showed the possibility to use donor-derived cell free DNA (ddcfDNA) for the early detection of graft rejection after introduction of nivolumab (26). This approach is minimally invasive and could help us detect early graft rejection and to adapt treatment accordingly. Other possible approaches to prevent rejection are the use of non-invasive biomarkers of the immune response induced by ICI: peripheral immune cell composition associated with graft tolerance and/or rejection, changes in the immune repertoire after administration of ICI, blood transcriptomics and urinary cell mRNA/chemokines profiling associated with acute rejection (for example, CXCL9, CD3ϵ, perforin, granzyme B, and IP-10) (27–32). With the advent of personalized medicine this approach is of interest to guide and adapt our treatment and to better inform our patients about the risks of graft rejection.

It has been hypothesized that a better clinical response in KTR may be associated with an increased risk of graft rejection. So far, in the KTR subgroup, graft rejection was not associated neither with a better tumoral response, nor with a higher mortality when compared to other SOTR. However maintaining low dose immunosuppression before start of ICI, the usage of mTORi and longer time elapsed since transplantation seem to be associated with a better ORR (17). ORR in our study is in line with most published reports and points to a possible benefit of cemiplimab in KTR suffering from advanced cSCC. All but one patient had preventive reduction in the dose of immunosuppression and most patients had kidney transplantation for at least 10 years.

The main limitations of our study are the retrospective design, the lack of control group and the low patient number. Only one patient developed graft rejection and our study was unable to identify the factors associated with rejection. However, the high ORR achieved with cemiplimab for advanced cSCC and the low number of graft rejection in our case series are encouraging. An interdisciplinary approach between oncologist and nephrologist is of utmost importance to guide treatment decisions and patient follow-up. Patients should be aware of the risk of graft rejection, but the present case series confirms and extends the data from recent literature regarding the good tumor response in advanced cSCC in KTR treated with ICI, with limited risk of graft rejection. We do however acknowledge the need of good designed prospective studies to search for biomarkers of rejection and/or tumor response, in order to guide our patients concerning the risk/benefit ratio of these treatments. Our center started a multicenter and international project to study allograft rejection in this particular patient population in search of the mechanisms and potential predictive markers of rejection before and during ICI treatment in KTR.

## Conclusion

The present case series shows that in KTR with advanced cSCC who failed to respond to conventional treatment, the use of cemiplimab is associated with a good ORR of 42.8% with low risk of graft rejection (14.3%) and irAE. Cemiplimab might be a promising treatment for patients with advanced cSCC and could be considered as second-line treatment in KTR with difficult to treat cSCC.

## Data availability statement

The raw data supporting the conclusions of this article will be made available by the authors, without undue reservation.

## Ethics statement

The studies involving human participants were reviewed and approved by Comité d’éthique de l’Hôpital Erasme. Written informed consent for participation was not required for this study in accordance with the national legislation and the institutional requirements.

## Author contributions

TM collected the data and wrote the manuscript. All authors contributed to the article and approved the submitted version.
