# Corrigendum: Cemiplimab for advanced cutaneous squamous cell carcinoma in kidney transplant recipients

**DOI:** 10.3389/fneph.2022.1129134

**Published:** 2023-01-05

**Authors:** T. Van Meerhaeghe, J.F. Baurain, O. Bechter, C. Orte Cano, V. Del Marmol, A. Devresse, P. Doubel, M. Hanssens, R. Hellemans, D. Lienard, A. Rutten, B. Sprangers, A. Le Moine, S. Aspeslagh

**Affiliations:** ^1^ Department of Nephrology, Hôpital Erasme – Université Libre de Bruxelles (ULB), Brussels, Belgium; ^2^ Department of Oncology, Clinique Universitaire Saint-Luc – Université Catholique de Louvain (UCLouvain), Brussels, Belgium; ^3^ Department of Oncology, Universitair Ziekenhuis (UZ) Leuven – Katholieke Universiteit Leuven (KUL), Leuven, Belgium; ^4^ Department of Dermatology, Hôpital Erasme – Université Libre de Bruxelles (ULB), Brussels, Belgium; ^5^ Department of Nephrology, Clinique Universitaire Saint-Luc – Université Catholique de Louvain (UCLouvain), Brussels, Belgium; ^6^ Department of Nephrology, Academisch Ziekenhuis (AZ) Groeninge, Kortrijk, Belgium; ^7^ Department of Oncology, Academisch Ziekenhuis (AZ) Groeninge, Kortrijk, Belgium; ^8^ Departement of Nephrology, Universitair Ziekenhuis (UZ) Antwerpen, Antwerpen, Belgium; ^9^ Department of Oncology, GasthuisZuster, Antwerpen, Belgium; ^10^ Department of Nephrology, Universitair Ziekenhuis (UZ) Leuven – Katholieke Universiteit Leuven (KUL), Leuven, Belgium; ^11^ Department of Oncology, Universitair Ziekenhuis (UZ) Brussel – Vrije Universiteit Brussel (VUB), Brussels, Belgium

**Keywords:** cemiplimab, kidney transplant recipients, cutaneous squamous cell carcinoma, immune checkpoint inhibitors, rejection

In the published article, there was a mistake in the **Funding** statement. The following statement should be removed: “TM declares that this study received funding from Sanofi and Bristol Myers Squibb. The funders were not involved in the study design, collection, analysis, interpretation of data, the writing of this article or the decision to submit it for publication”. The correct statement appears below.

